# Neddylation is required for herpes simplex virus type I (HSV-1)-induced early phase interferon-beta production

**DOI:** 10.1038/cmi.2015.35

**Published:** 2015-05-11

**Authors:** Xueying Zhang, Zhenjie Ye, Yujun Pei, Guihua Qiu, Qingyang Wang, Yunlu Xu, Beifen Shen, Jiyan Zhang

**Affiliations:** 1 Department of Molecular Immunology, Institute of Basic Medical Sciences, 27 Taiping Road, Beijing 100850, P. R. China; 2 Laboratory of Snake Venom, Fujian Medical University, 88 Jiaotong Road, Fuzhou 350004, P. R. China.

**Keywords:** innate immunity, HSV-1, IFN-β, NF-κB, IRF3

## Abstract

Type I interferons such as interferon-beta (IFN-β) play essential roles in the host innate immune response to herpes simplex virus type I (HSV-1) infection. The transcription of type I interferon genes is controlled by nuclear factor-κB (NF-κB) and interferon regulatory factor (IRF) family members including IRF3. NF-κB activation depends on the phosphorylation of inhibitor of κB (IκB), which triggers its ubiqitination and degradation. It has been reported that neddylation inhibition by a pharmacological agent MLN4924 potently suppresses lipopolysaccharide (LPS)-induced proinflammatory cytokine production with the accumulation of phosphorylated IκBα. However, the role of neddylation in type I interferon expression remains unknown. Here, we report that neddylation inhibition with MLN4924 or upon UBA3 deficiency led to accumulation of phosphorylated IκBα, impaired IκBα degradation, and impaired NF-κB nuclear translocation in the early phase of HSV-1 infection even though phosphorylation and nuclear translocation of IRF3 were not affected. The blockade of NF-κB nuclear translocation by neddylation inhibition becomes less efficient at the later time points of HSV-1 infection. Consequently, HSV-1-induced early phase IFN-β production significantly decreased upon MLN4924 treatment and UBA3 deficiency. NF-κB inhibitor JSH-23 mimicked the effects of neddylation inhibition in the early phase of HSV-1 infection. Moreover, the effects of neddylation inhibition on HSV-1-induced early phase IFN-β production diminished in the presence of NF-κB inhibitor JSH-23. Thus, neddylation contributes to HSV-1-induced early phase IFN-β production through, at least partially, promoting NF-κB activation.

## INTRODUCTION

Herpes simplex virus type I (HSV-1) is a common human pathogen that establishes a life-long infection in about 80% of adults.^1–2^ HSV-1 typically causes oral lesions but occasionally causes blindness and fatal sporadic encephalitis.^1–2^ Type I interferons such as interferon-beta (IFN-β) play essential roles in the host innate immune response to HSV-1 infection.^2^ The transcription of type I interferon genes is controlled by nuclear factor-κB (NF-κB) and interferon regulatory factor (IRF) family members including IRF3.^3^ In unstimulated cells, NF-κB is sequestered in the cytoplasm by inhibitor of κB (IκB) proteins. Recognition of viral DNA leads to the activation of the IκB kinase (IKK)-related kinases, TANK-binding kinase 1 (TBK1), and IKKi, which triggers the phosphorylation of IκB and IRF proteins.^3^ Phosphorylation of IκB proteins leads to ubiquitin-mediated degradation, thereby promoting nuclear translocation of NF-κB, whereas phosphorylation of IRF3 induces its homodimerization and accumulation into the nucleus.^3^ In the nucleus, the activated transcription factors induce gene transcription.

The ubiquitination of IκB proteins is mediated by an ubiquitin E3 ligase complex comprised of multiple components, including Cullin1.^4^ The activity of this ubiquitin E3 ligase complex depends on Cullin1 neddylation.^5–6^ Neddylation occurs in a multistep enzymatic process in which NEDD8, a highly conserved ubiquitin-like protein, is covalently conjugated to target proteins.^7^ NEDD8-activating enzyme (NAE) is the heterodimer of catalytic subunit ubiquitin-like modifier activating enzyme 3 (UBA3) and amyloid precursor protein binding protein-1 (APPBP1).^7^ It has been demonstrated that NAE-specific inhibitor MLN4924 potently inhibits lipopolysaccharide (LPS)-induced proinflammatory cytokine production with the accumulation of phosphorylated IκBα.^8,9,10,11^ However, the role of neddylation in the production of type I interferons has not been reported. In this work, we report that neddylation is essential for HSV-1-induced early phase IFN-β production through, at least partially, promoting NF-κB activation.

## MATERIALS AND METHODS

### Mice

Mice were generated at Shanghai Research Center for Biomodel Organisms (Shanghai, China). Frt-flanked neomycin cassette was introduced immediately after exon 3 (121-bp) of the *UBA3* gene. *LoxP* sites were situated right before exon 3 and after the Frt-Neo-Frt sequence. The construct was electroporated into 129S_V_/E_V_ embryonic stem (ES) cells. After neomycin selection, the ES cells were injected into foster mothers of C57 BL/6J background to create chimeric mice that transmitted the mutated *UBA3* allele through the germ line. The mice were backcrossed to the C57 BL/6J strain (Jackson Laboratory). Mice homozygous for a *UBA3* conditional allele (*UBA3*^F/F^) were crossed with mice (Jackson Laboratory) expressing the lysozyme promoter-driven *cre* recombinase (*Lyz2-Cre*) gene. *UBA3*^F/F; Lyz2-Cre^ (named as *UBA3*^Δmye^) mice were kept in the animal facility of Institute of Biotechnology (20 Dongdajie, Beijing 100071, P. R. China). The genotypes were determined by PCR of tissue-extracted DNA. PCR primers for the *UBA3* conditional allele were: 5′- catctttccaacttgggaggagcc-3′ (forward) and 5′-gggtccagaccgctcgaggaact-3′ (reverse), for the *Lyz2-Cre* gene were: 5′-gcctgcattaccggtcgatgc-3′ (forward) and 5′- cagggtgttataagcaatccc-3′ (reverse). Animals were handled in accordance with institutional guidelines. Studies were performed with 8- to 12-week-old mice. Animal experiments were approved by the Institutional Animal Care and Use Committee.

### Reagents

Antibodies against phospho-IκBα, phospho-IRF3, NEDD8, and UBA3 were purchased from Cell Signaling Technology (Beverly, MA, USA). Antibodies against IκBα and actin and NF-κB inhibitor JSH-23 (dissolved in dimethylsulphoxide, DMSO) were purchased from Santa Cruz (Santa Cruz, CA, USA). Antibody against p65 was from Epitomics (Burlingame, CA). Antibody against IRF3 was from Abclonal (Cambridge, MA, USA). FBS was from HyClone Laboratories (Logan, UT, USA). M-CSF was from Cetus (Emeryville, CA, USA). TRIzol reagent, Moloney murine leukemia virus reverse transcriptase, and oligo(dT) primer were from Invitrogen (Carlsbad, CA, USA).

### Induction of bone marrow-derived macrophages

Bone marrow-derived macrophages (BMMs) were generated by flushing bone marrow cells from femurs and tibiae of mice. Cells were cultured in RPMI 1640 medium containing 15% (v/v) FBS, 2 mM L-glutamine, 100 U/ml penicillin, 100 μg/ml streptomycin, and 50 μM β-mercaptoethanol with 100 ng/ml M-CSF for 7 d.

### Virus preparation and infection

HSV-1 virus (Kos strain, Chinese Academy of Medical Sciences, Beijing) was propagated in HeLa cells. At the peak of cytopathogenic effect, the supernatant was harvested and clarified by centrifugation. The supernatant was then aliquoted and stored at −80°C. BMMs were infected with HSV-1 (5 M.O.I.) for 1 h; cells were washed with PBS and cultured in fresh media for the indicated periods of time.

### Immunoblotting

BMMs were washed with PBS and harvested with a cell scraper (Costars, Cambridge, MA, USA) in ice-cold lysis buffer (0.5% NP-40, 20 mM Tris-Cl, pH 7.6, 250 mM NaCl, 3 mM EDTA, 3 mM EGTA, 1 mM sodium orthovanadate, 1 mM DTT, 10 mM PNPP, 10 μg/ml aprotinin). Cell lysates were resolved by SDS-PAGE before being transferred to nitrocellulose membranes. Nitrocellulose membranes were then incubated with 5% (w/v) nonfat dry milk in washing buffer (20 mM Tris-Cl, pH 7.6, 150 mM NaCl, and 0.1% Tween 20) for 1 h at 37°C to block nonspecific protein binding. Primary antibodies (1:1000) were diluted in washing buffer containing 3% BSA and applied to the membranes for overnight at 4°C. After extensive washing, the membranes were incubated with goat anti-rabbit IgG-HRP (diluted up to 1:2500 in washing buffer containing 5% (w/v) nonfat dry milk) for 1 h at room temperature. Following washing, immunoreactive bands were visualized by the ECL Chemiluminescence Kit.

### Immunofluorescence

BMMs grown on slides were fixed with 4% (w/v) paraformaldehyde in PBS for 10 min at room temperature and then permeabilized with 0.5% Triton X-100 in PBS for 15 min. The nonspecific sites were blocked by incubation with 1% bovine serum albumin in PBS for 30 min at room temperature. Cells were then rinsed in PBS containing 0.05% Tween 20 for 5 min and incubated with rabbit monoclonal antibody against p65 diluted in blocking buffer for 1 h at room temperature or overnight at 4°C. After being washed for three times in PBS containing 0.05% Tween 20, the cells were incubated with TRITC-conjugated goat anti-rabbit IgG for 45 min at room temperature. The cells were washed again as stated above, incubated with 1 μg/mL 4′,6-diamidine-2-phenylindole (DAPI),and then observed under a laser scanning confocal microscopy (RADIANCE 2100; Bio-Rad, Hercules, CA, USA).

## ELISA

Cell supernatants were collected at indicated time points after HSV-1 treatment. The concentration of IFN-α and IFN-β was measured with ELISA kits purchased from Biolegend (San Diego, CA, USA) and PBL Assay Science (Piscataway, NJ, USA), respectively, according to the manufacturers' protocols.

### Quantitative real-time reverse-transcriptase PCR

RNA was extracted by using TRIzol reagent. cDNA was derived from 1 μg total RNA by reverse transcription using Moloney murine leukemia virus reverse transcriptase and oligo(dT) primer. Quantitative PCR was performed by SYBR Premix EX TaqII (Takara) using a CFX96 Real-Time System (Bio-Rad). PCR conditions for all assays were 94°C for 30 seconds, followed by 40 cycles of amplification (94°C for 5 seconds, 60°C for 30 seconds, and 72°C for 30 seconds). GAPDH mRNA was used to normalize RNA inputs. Primers for GAPDH were: 5′-ggcaaattcaacggcacagt-3′ (forward) and 5′-agatggtgatgggcttccc-3′ (reverse). Primers for IFN-β were: 5′-atgagtggtggttgcaggc-3′ (forward) and 5′-tgacctttcaaatgcagtagattca-3′ (reverse).

### Statistical analysis

The data were shown as mean ± standard deviations (SD). Student's *t*-test was used to compare the difference between the two groups. The difference was considered statistically significant when *p* < 0.05.

## RESULTS

### Neddylation inhibition with MLN4924 leads to impaired NF-κB activation in the early phase of HSV-1 infection

**Figure 1:**
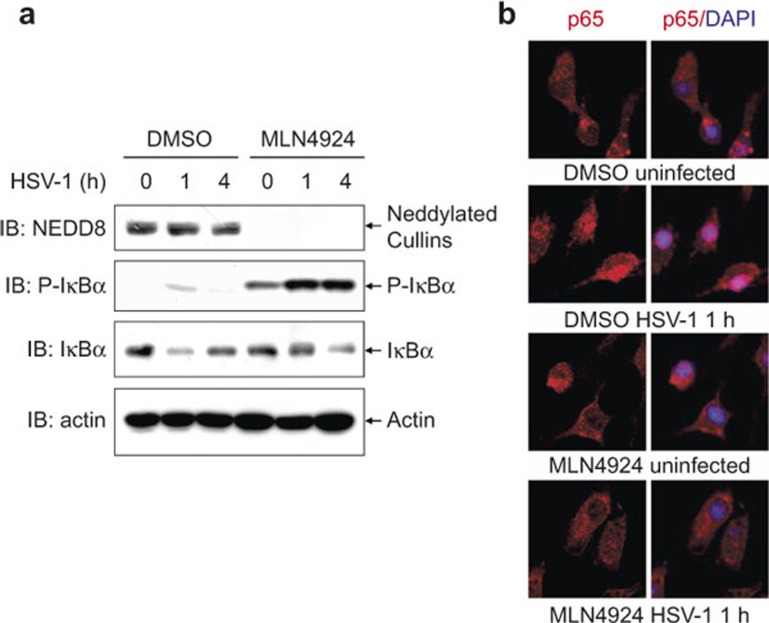
Neddylation inhibition with MLN4924 leads to impaired NF-κB activation in the early phase of HSV-1 infection. After BMMs were pretreated with 0.1 μM MLN4924 or DMSO of equal volume for 30 min, cells were infected with HSV-1 for 0, 1, and 4 h. (**a**) Cell lysates were harvested and subjected to immunoblotting (IB) analysis with the indicated antibodies. (**b**) The subcellular localization of p65 subunit of NF-κB (red) was revealed by indirect immunofluorescence staining with a p65-specific antibody. Nuclei were counterstained for DNA by DAPI (blue).

To explore whether neddylation affects NF-κB activation in response to HSV-1 infection, BMMs were pretreated with 0.1 μM MLN4924 for 30 min, infected with HSV-1 for 0, 1, and 4 h, and subjected to immunoblotting analysis with specific antibodies. As shown in Figure 1a, 0.1 μM MLN4924 significantly abrogated the neddylation of Cullins. IκBα degradation in response to HSV-1 infection was delayed in MLN4924-pretreated BMMs, which was associated with significant accumulation of phosphorylated IκBα (Figure 1a). Consistently, indirect immunofluorescence microscopy revealed that HSV-1-induced nuclear translocation of p65 subunit of NF-κB was impaired in MLN4924-pretreated BMMs (Figure 1b). Thus, neddylation inhibition with MLN4924 leads to impaired NF-κB activation in the early phase of HSV-1 infection.

### Neddylation inhibition with MLN4924 does not affect IRF3 activation in the early phase of HSV-1 infection

**Figure 2:**
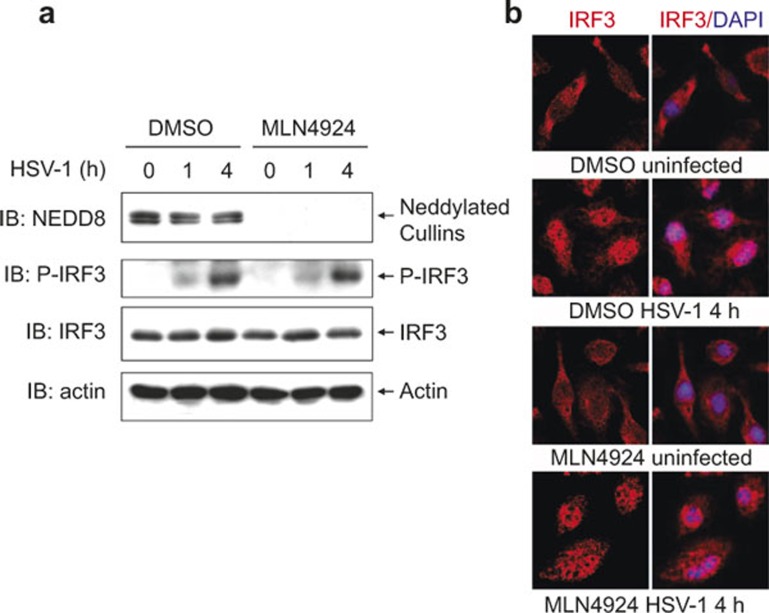
Neddylation inhibition with MLN4924 does not affect IRF3 activation in the early phase of HSV-1 infection. After BMMs were pretreated with 0.1 μM MLN4924 or DMSO of equal volume for 30 min, cells were infected with HSV-1 for 0, 1, and 4 h. (**a**) Cell lysates were harvested and subjected to immunoblotting analysis with the indicated antibodies. (**b**) The subcellular localization of IRF3 (red) was revealed by indirect immunofluorescence staining with an IRF3-specific antibody. Nuclei were counterstained for DNA by DAPI (blue).

The transcription of type I interferon genes is controlled by IRF family members including IRF3 as well as NF-κB.^3^ Next, we examined whether neddylation inhibition with MLN4924 affects IRF3 activation in the early phase of HSV-1 infection. Immunoblotting analysis revealed that HSV-1-induced IRF3 phosphorylation remained unchanged with MLN4924 pretreatment (Figure 2a). Moreover, indirect immunofluorescence microscopy revealed that HSV-1-induced nuclear translocation of IRF3 was not affected (Figure 2b). These data suggest that neddylation inhibition does not affect IRF3 activation in the early phase of HSV-1 infection and exclude the possibility that neddylation inhibition disturbs the upstream events of NF-κB activation.

### HSV-1-induced early phase NF-κB activation is impaired upon UBA3 deficiency

**Figure 3:**
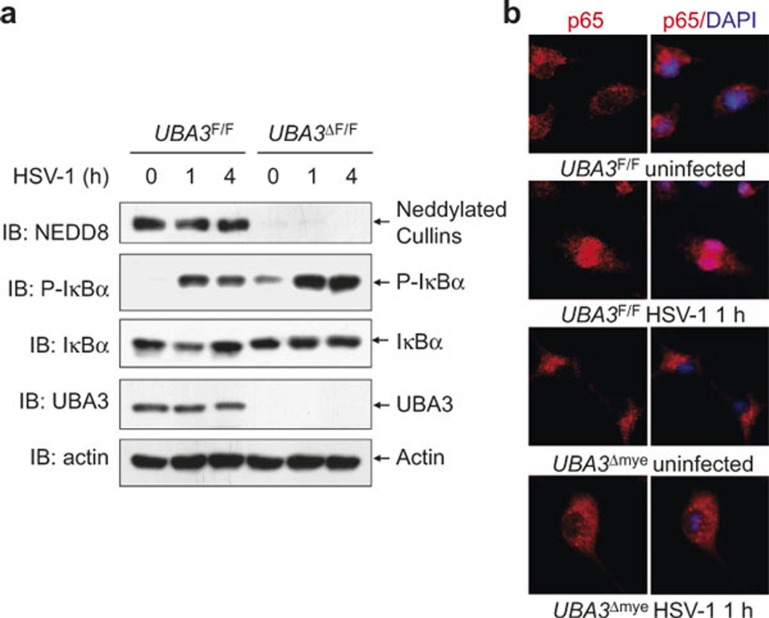
HSV-1-induced early phase NF-κB activation is impaired upon UBA3 deficiency. BMMs from *UBA3*^F/F^ and *UBA3*^ΔMye^ mice were infected with HSV-1 for 0, 1, 4 h. (**a**) Cell lysates were harvested and subjected to immunoblotting analysis with the indicated antibodies. (**b**) The subcellular localization of p65 subunit of NF-κB (red) was revealed by indirect immunofluorescence staining with a p65-specific antibody. Nuclei were counterstained for DNA by DAPI (blue).

Our previous data suggest that neddylation inhibition with MLN4924 leads to impaired NF-κB activation in the early phase of HSV-1 infection without disturbing the upstream molecular events. To confirm the defective early phase NF-κB activation upon neddylation inhibition, we cultured BMMs from mice homozygous for a *UBA3* conditional allele (*UBA3*^F/F^) and *UBA3*^F/F; Lyz2-Cre^ (named as *UBA3*^Δmye^) mice. BMMs were infected with HSV-1 for 0, 1, and 4 h, and subjected to immunoblotting analysis with specific antibodies. As shown in Figure 3a, UBA3 deficiency significantly abrogated the neddylation of Cullins. Furthermore, HSV-1-induced IκBα degradation was delayed upon UBA3 deficiency, which was associated with significant accumulation of phosphorylated IκBα (Figure 3a). Consistently, indirect immunofluorescence microscopy revealed that HSV-1-induced nuclear translocation of p65 subunit of NF-κB was impaired upon UBA3 deficiency (Figure 3b). These data confirm that neddylation inhibition leads to impaired NF-κB activation in the early phase of HSV-1 infection.

### HSV-1-induced early phase IRF3 activation remains unchanged upon UBA3 deficiency

**Figure 4:**
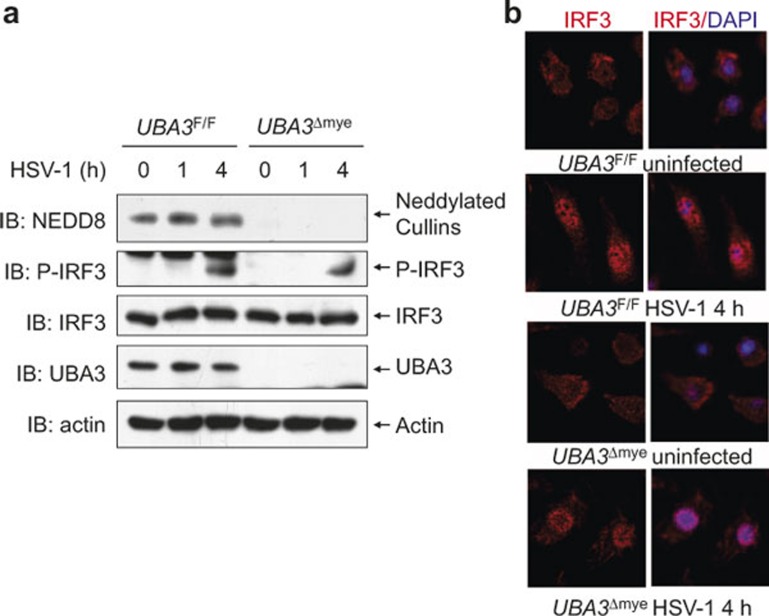
HSV-1-induced early phase IRF3 activation remains unchanged upon UBA3 deficiency. BMMs from *UBA3*^F/F^ and *UBA3*^ΔMye^ mice were infected with HSV-1 for 0, 1, and 4 h. (**a**) Cell lysates were harvested and subjected to immunoblotting analysis with the indicated antibodies. (**b**) The subcellular localization of IRF3 (red) was revealed by indirect immunofluorescence staining with an IRF3-specific antibody. Nuclei were counterstained for DNA by DAPI (blue).

Next, we examined whether UBA3 deficiency affects IRF3 activation in the early phase of HSV-1 infection. Immunoblotting analysis revealed that HSV-1-induced IRF3 phosphorylation remained unchanged upon UBA3 deficiency (Figure 4a). Moreover, indirect immunofluorescence microscopy revealed that HSV-1-induced nuclear translocation of IRF3 was not affected (Figure 4b). These data confirm that neddylation inhibition does not affect early phase IRF3 activation in response to HSV-1 infection.

### The suppression of NF-κB nuclear translocation upon neddylation inhibition becomes less efficient at the later time points of HSV-1 infection

**Figure 5:**
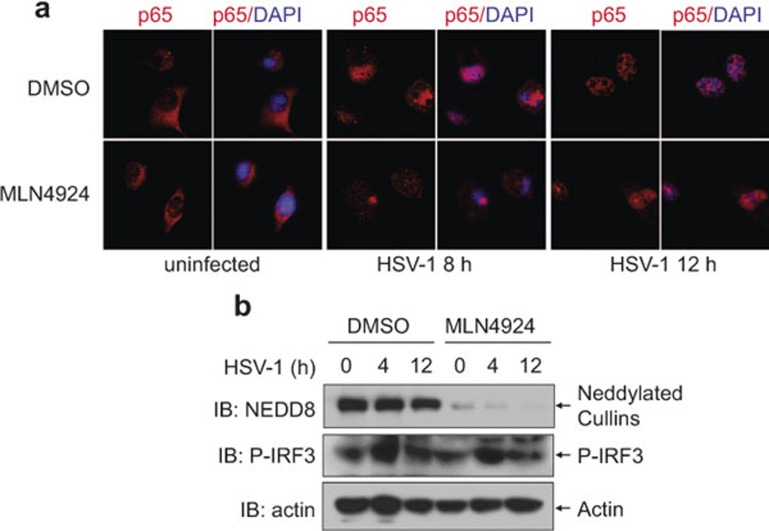
The suppression of NF-κB nuclear translocation upon neddylation inhibition becomes less efficient at the later time points of HSV-1 infection. After BMMs were pretreated with 0.1 μM MLN4924 or DMSO of equal volume for 30 min, cells were infected with HSV-1 for the indicated periods of time. (**a**) The subcellular localization of p65 subunit of NF-κB (red) was revealed by indirect immunofluorescence staining with a p65-specific antibody. Nuclei were counterstained for DNA by DAPI (blue). (**b**) Cell lysates were harvested and subjected to immunoblotting analysis with the indicated antibodies.

Our previous data indicate that neddylation inhibition leads to delayed, but not totally blocked, IκBα degradation in response to HSV-1 infection (Figures 1a and 3a). To further examine the extent of impaired NF-κB activation, we checked p65 nuclear translocation in response to HSV-1 infection at later time points with or without MLN4924 pretreatment. As shown in Figure 5a, p65 remained in the nucleus of control BMMs 8–12 h after HSV-1 infection (Figure 5a). p65 equally distributed in the cytoplasm and nucleus of MLN4924-pretreated BMMs 8 h after HSV-1 infection (Figure 5a). After 12 h of HSV-1 infection, p65 showed predominantly nuclear localization even in the presence of MNL4924 (Figure 5a). Thus, neddylation inhibition leads to delayed, but not totally blocked, NF-κB nuclear translocation in response to HSV-1 infection. On the other hand, IRF3 phosphorylation decreased to the basal level in control BMMs 12–24 h after HSV-1 infection, which was unaffected in the presence of MLN4924 (Figure 5b and data not shown). Therefore, neddylation inhibition does not affect IRF3 activation even at the later time points of HSV-1 infection.

### Neddylation inhibition dampens HSV-1-induced early phase IFN-β production

**Figure 6:**
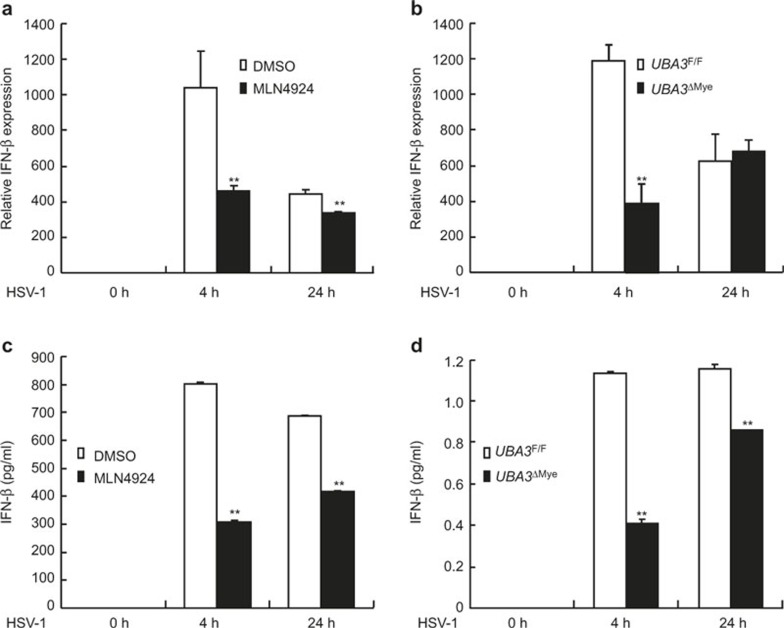
Neddylation inhibition dampens HSV-1-induced early phase IFN-β production. BMMs were pretreated with 0.1 μM MLN4924 or DMSO of equal volume for 30 min (**a,c**) or BMMs were cultured from *UBA3*^F/F^ and *UBA3*^ΔMye^ mice (**b,d**). Then BMMs were infected with HSV-1 for the indicated periods of time. Relative IFN-β mRNA expression was measured by qRT-PCR (**a,b**) and IFN-β concentration in the supernatants was measured with ELISA (**c,d**). **p < 0.01.

Since neddylation inhibition upon MLN4924 pretreatment or UBA3 deficiency leads to impaired NF-κB activation in the early phase of HSV-1 infection, we then explored whether neddylation inhibition affected IFN-β expression with quantitative real-time reverse-transcriptase PCR. As expected, the level of IFN-β mRNA was significantly upregulated 4 h after HSV-1 infection, which dropped about 50% 24 h after HSV-1 infection (Figure 6a,b). A measure of 0.1 μM MLN4924 pretreatment for 30 min led to about 50% reduction of IFN-β mRNA 4 h after HSV-1 infection (Figure 6a). The inhibitory effect diminished 24 h after HSV-1 infection (Figure 6a). UBA3 deficiency exhibited similar effects on HSV-1-induced upregulation of IFN-β mRNA (Figure 6b).

Next, we tried to confirm neddylation inhibition dampens IFN-β expression with ELISA. As expected, the level of IFN-β in the supernatant of BMMs was significantly upregulated 4 h after HSV-1 infection, which remained stable 24 h after HSV-1 infection (Figure 6c,d). 0.1 μM MLN4924 pretreatment for 30 min led to about two-thirds reduction of IFN-β production 4 h after HSV-1 infection (Figure 6c). The inhibitory effect became less efficient 24 h after HSV-1 infection (Figure 6c). UBA3 deficiency exhibited similar effects on HSV-1-induced upregulation of IFN-β secretion (Figure 6d).

### NF-κB inhibitor JSH-23 dampens HSV-1-induced early phase IFN-β production

Our previous data indicate that neddylation inhibition leads to impaired NF-κB activation in the early phase of HSV-1 infection and neddylation inhibition dampens HSV-1-induced early phase IFN-β production. Because neddylation inhibition does not affect IRF3 activation, most likely neddylation inhibition dampens HSV-1-induced early phase IFN-β production through delaying NF-κB activation. To explore whether NF-κB is essential for HSV-1-induced early phase IFN-β production, we pretreated BMMs with different doses of JSH-23, a specific inhibitor of NF-κB nuclear translocation and transcriptional activity,^12^ before BMMs were subjected to HSV-1 infection for 4 h. ELISA revealed that JSH-23 potently inhibited HSV-1-induced early phase IFN-β production at the dose as low as 2.5 μM and the inhibitory effect became more significant when the dose increased (Figure 6a). However, JSH-23 at the dose higher than 5 μM led to decreased cell number after HSV-1 infection possibly due to apoptosis (Figure 6b). Therefore, we chose the dose of 5 μM to test whether JSH-23 blocked HSV-1-induced p65 nuclear translocation. Consistent with the previous report, indirect immunofluorescence microscopy revealed that HSV-1-induced nuclear translocation of p65 subunit of NF-κB was impaired in JSH-23-pretreated BMMs (Figure 6c).

### The effects of neddylation inhibition on HSV-1-induced early phase IFN-β production diminishes in the presence of NF-κB inhibitor JSH-23

**Figure 7:**
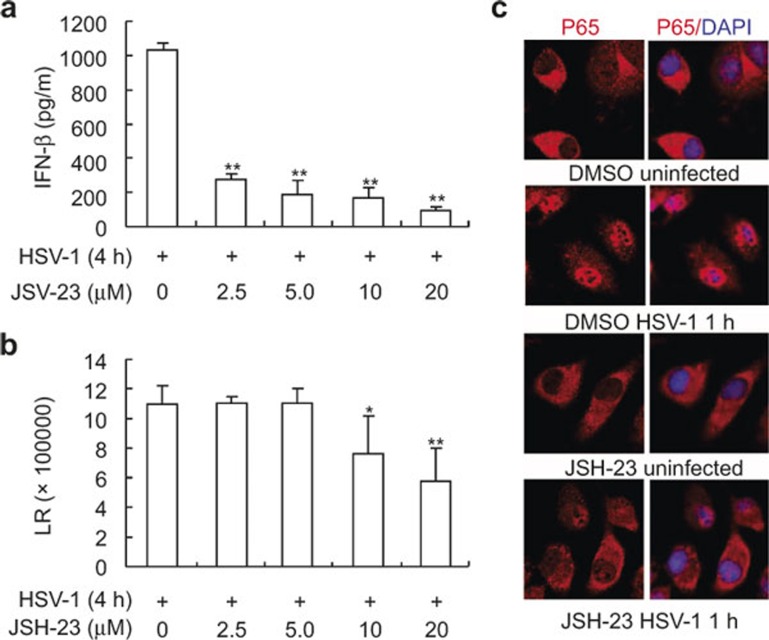
NF-κB inhibitor JSH-23 inhibits HSV-1-induced IFN-β production. (**a,b**) BMMs were pretreated with various doses of JSH-23 for 30 min, followed by HSV-1 infection. 4 h later, IFN-β concentration in the supernatants was measured with ELISA (**a**) and cell viability was measured with ATPlite assay. LR, luminescence reading (**b**). (**c**) After BMMs were pretreated with 5 μM JSH-23 or DMSO of equal volume for 30 min, cells were infected with HSV-1 for 1 h. The subcellular localization of p65 subunit of NF-κB (red) was revealed by indirect immunofluorescence staining with a p65-specific antibody. Nuclei were counterstained for DNA by DAPI (blue). *p < 0.05, **p < 0.01.

To confirm the key role of NF-κB in the effects of neddylation inhibition on HSV-1-induced early phase IFN-β production, we pretreated BMMs with 5 μM JSH-23 before BMMs were subjected to HSV-1 infection for 4 h in the presence of 0.1 μM MLN4924 or upon UBA3 deficiency. ELISA revealed that JSH-23 alone showed more profound inhibition on HSV-1-induced early phase IFN-β production than MLN4924 pretreatment or UBA3 deficiency (Figure 7a,b). Because neddylation inhibition and JSH-23 pretreatment blocked NF-κB nuclear translocation in the early phase of HSV-1 infection with similar efficiency, the more profound inhibitory effect of JSH-23 on HSV-1-induced early phase IFN-β production might be attributed to its additional ability to block NF-κB transcriptional activity. The effects of neddylation inhibition on HSV-1-induced early phase IFN-β production diminishes in the presence of NF-κB inhibitor JSH-23 (Figure 7a,b). Thus, neddylation contributes to HSV-1-induced early phase IFN-β production through, at least partially, promoting NF-κB activation.

### Discussion

Double stranded DNA virus HSV-1 is a very common human pathogen causing a number of diseases. Macrophages and their produced cytokines contribute to the innate immune response against HSV-1 infection and further behave as an important link between innate and adaptive immunity.^13^ On the other hand, the importance of a timely and measured type I interferon response in the control of HSV-1 infection is well-established.^14^ Here, we used BMMs to study how neddylation might be involved in the production of type I interferon IFN-β triggered by HSV-1 infection. We show that neddylation is essential for the efficient early-phase IFN-β production in response to HSV-1 infection. To our knowledge, this is the first report about the role of neddylation in type I interferon production.

Neddylation, a type of protein post-translational modification similar to ubiquitination, regulates diverse biological processes by affecting subcellular localization, stability, and function of target proteins.^7^ The role of neddylation in cancer cell biology has been extensively investigated with NAE-specific inhibitor MLN4924 as the major tool.^15^ The NF-κB pathway has been demonstrated as a key target for MLN4924 to exert its effects because ubiquitination of phosphorylated IκB and its subsequent degradation depend on neddylation.^8,9,10,11^ Here, we report that neddylation is essential for NF-κB activation in the early phase of HSV-1 infection. However, the blockade of NF-κB nuclear translocation by neddylation inhibition becomes less efficient at the later time points of HSV-1 infection. This might be attributed to delayed, but not totally blocked, HSV-1-induced IκBα degradation upon neddylation inhibition. The underlying molecular mechanisms remain to be explored.

It is well established that activation of the IFN-β promoter requires the binding of not only IRF proteins but also NF-κB.^3^ Our data further confirm an essential role for NF-κB in the efficient production of IFN-β, particularly in the early phase of HSV-1 infection. Consistent with our finding that neddylation is essential for NF-κB activation in the early phase of HSV-1 infection, our data show that neddylation promotes HSV-1-induced early phase IFN-β production. The inhibitory effect became less efficient 24 h after HSV-1 infection in MLN4924 pretreated or UBA3 deficient BMMs. Since IRF3 activation in the early phase and at the later time points of HSV-1 infection was not affected by MLN4924, the dampened inhibitory effect should result from the partial recovery of NF-κB nuclear translocation.

Besides IFN-β, another major member of the type I family of interferons is IFN-α.^13,14^ We also put some efforts to examine how neddylation might affect HSV-1-induced IFN-α production. Similar to the data about IFN-β, MLN4924 potently inhibited HSV-1-induced early phase IFN-α production (Supplementary Figure 1). However, the inhibitory effect became less efficient 24 h after HSV-1 infection (Supplementary Figure 1). MLN4924 is already in the clinic trail for cancer therapy.^16^ Our findings of inhibition of HSV-1-induced type I interferon production by MLN4924 suggest that DNA virus infection might be a potential threat during the administration of this drug.

## Figures and Tables

**Figure 8:**
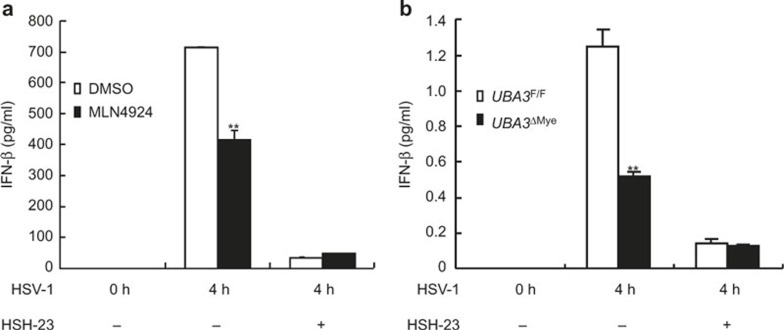
The effects of neddylation inhibition on HSV-1-induced early phase IFN-β production diminishes in the presence of NF-κB inhibitor JSH-23. (**a**) BMMs were pretreated with 0.1 μM MLN4924 and/or 30 μM JSH-23 or DMSO of equal volume for 30 min. or (**b**) BMMs from *UBA3*^F/F^ and *UBA3*^ΔMye^ mice were pretreated with 30 μM JSH-23 or DMSO of equal volume for 30 min. Then BMMs were infected with HSV-1 for the indicated periods of time cells and IFN-β concentration in the supernatants was measured with ELISA. **p < 0.01.
