# Supplementary figures and images for: Neddylation is required for herpes simplex virus type I (HSV-1)-induced early phase interferon-beta production
Source: Cell Mol Immunol. 2015 May 11;13(5):578–83. doi: 10.1038/cmi.2015.35 (PMC5037273; doi:10.1038/cmi.2015.35)

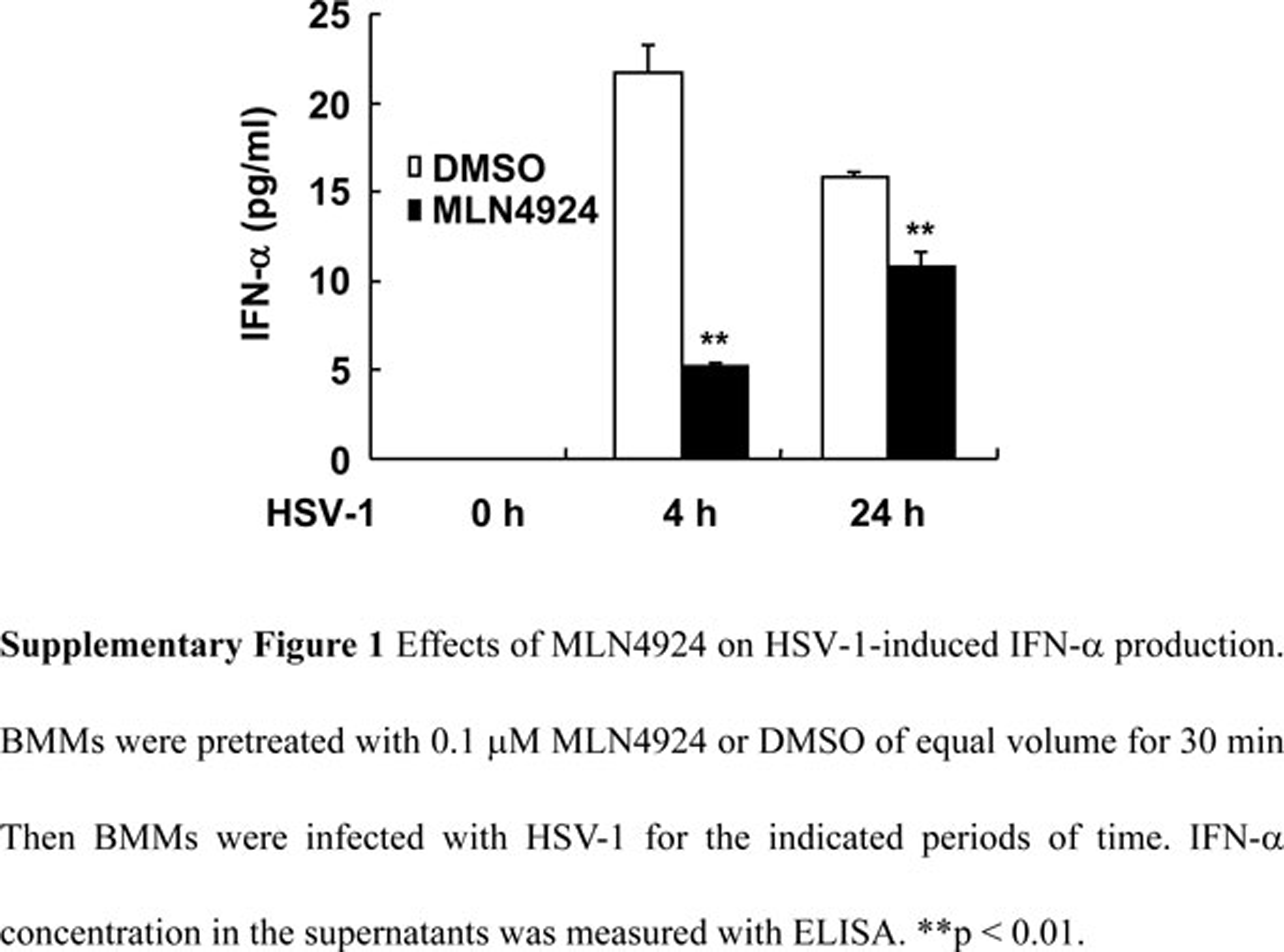

Supplement: Supplementary Figure 1 [file cmi201535x1.tif]
